# Debt Problem of One Partner and Depressive Morbidity in the Other: A 2-Year Follow-up Register Study of Different-Sex Couples in Sweden

**DOI:** 10.1007/s10834-022-09817-4

**Published:** 2022-02-07

**Authors:** Yerko Rojas

**Affiliations:** School of Social Sciences, Södertörn University, 141 89 Huddinge, Sweden

**Keywords:** Depression, Antidepressants, Financial indebtedness, Family, Linked lives, Sweden

## Abstract

This study sets out to examine whether depressive morbidity varies by status of financial indebtedness of a spouse or cohabiting partner. For this purpose, individuals aged between 20 and 60 with a different-sex spouse/cohabiting partner with a registration date for a debt at the Swedish Enforcement Authority (SEA) during 2017 (n = 6979) are followed-up for a 2-year period for prescriptions of antidepressants and compared with a sample from the general Swedish population (n = 29,708). The analysis is based on penalized maximum likelihood logistic regressions. Both women and men were more likely to suffer from depressive morbidity if the spouse/cohabiting partner had been registered at the SEA in 2017 and was still active for a debt in the SEA’s register in 2018 (OR 1.31 and OR 1.57, respectively), irrespective of their own health, employment, socioeconomic status, and other background variables. This also held true for men if a wife/cohabiting partner had been registered at the SEA in 2017 but was no longer active for a debt in the SEA’s register in 2018 (OR 1.29). For women, on the other hand, only those with no history (11-year period) of prescription of psychotropic medications were also at an enhanced risk of depressive morbidity if a husband/cohabiting partner had gone from being registered for a debt at the SEA in 2017, to not being registered as active for a debt in the SEA’s register in 2018 (OR 1.24). The results reinforce the importance of acknowledging that negative effects of financial indebtedness extend beyond the individual debtor.

## Introduction

Depressive disorders are a leading cause of disability globally (Jensen et al., 2019); even milder depressive symptoms are known to cause distress and impairment (Cuijpers et al., 2004). Up until recently, financial indebtedness, that is, being in debt and not having the capacity to pay what is owed (Krumer-Nevo et al., 2017), was rather uncontestably thought to be a source of this form of ill health (Turunen & Hiilamo, 2014). However, it has been noted that selection of problem debt on the basis of poor psychological health accounts for much of the observed cross-sectional variation in depression between those with and without debt problems (Alegría et al., 2018; Gathergood, 2012). Furthermore, findings of a direct link between problem debt and depression have tended not to be supported when using an objective measure of indebtedness as opposed to self-reported ones (Bridges & Disney, 2010). These mixed findings raise the question of whether the more complex relations that have been derived from it, such as the relation between an individual’s indebtedness and the depression of their cohabiting partner or spouse—which is also the one to be tested in the present study—are still valid. This is especially so, considering that the evidence that exists for this particular relation is in practice based on cross-sectional, longitudinal spouse level, self-reported, or simply qualitative data (Bridges & Disney, 2010; Dew, 2008, 2011; Drentea, 2000; Thorne, 2010).

Although theories on how debt affects couple’s well-being are underdeveloped (Dew, 2007), the assumption that indebtedness, as an indicator of economic strain, is related to a spouse’s emotional distress, depression in particular, is a key one within the family stress theory. A theory that has been the basis for numerous studies (Dew & Yorgason, 2010; Drentea, 2000). However, re-testing the extent to which depression can be attributed to the financial indebtedness of a cohabiting partner or a spouse, using a type of data that has not been used previously, as in this case, large-scale individual-level prospective data with directly (otherwise known as objectively) assessed measures—including information on individuals’ history of mental illness—is not only relevant from an academic point of view. Policy wise, it would seem to be important to confirm the validity of the recommendation made by international health agencies (WHO, 2009), as late as in the wake of the financial crisis of 2007–2008, of needing to be aware of the detrimental mental health impact that unfavorable economic changes tend to have on others than the directly affected individual within a family. Not the least at a time when the COVID-19 pandemic is unleashing the largest contraction in economic activity since the Great Depression (UNDP, 2020), and debt constitutes an integral part of social life (Charbonneau & Hansen, 2014; Lazzarato, 2012).

By making use of the unique opportunity offered by the Microdata Online Access (MONA) system at Statistics Sweden to link and analyze data from different nationwide registers, this study has been able to follow the prescription of antidepressant medications among 20–60-year-old women and men who had a different-sex spouse or cohabiting partner, with a registration date for an unpaid debt in the Swedish Enforcement Authority (SEA) register during 2017. The study also employed a comparison group from the general Swedish population.

### Prescription of Antidepressants and Depressive Morbidity

Depressive morbidity is a term used to denote everything from depressive symptoms to major depressive disorders (Capuron et al., 2017; Ohayon & Schatzberg, 2003; Silva et al., 2014), and it is also a term that is used when analyzing prescriptions or use of antidepressants in a population (Leinonen et al., 2017). Part of the explanation is that antidepressants are primarily used to treat both moderate and severe depression (Forns et al., 2019), but they have also been found to be prescribed for milder forms of depression (OECD, 2014).

It should be noted that antidepressants are also used for a wide range of other conditions, such as anxiety, panic disorders, chronic pain, and sleep problems (Forns et al., 2019; Leinonen et al., 2017). However, non-psychic indications are typically more common in the elderly (Leinonen et al., 2017), and the fact that anxiety, panic issues, and/or sleeping problems might be part of what is also being captured in the measure is in line with the main theoretical assumption on which this study is based, namely, that financial indebtedness of a partner is psychologically distressing for the other partner. Furthermore, panic attacks and other symptoms of anxiety, as well as sleeping problems, are known to pre-date depressive episodes (Castriotta & Craske, 2014; Nutt et al., 2008).

### Cross-Over Effects of Indebtedness and Emotional Distress: The Family Stress Model

It is theoretically sound to assume that lives are lived interdependently and hence affect each other (e.g., the principle of linked lives (Elder et al., 2003)). Studies on how financial hardship influences families have often been formulated through the family stress theory (Ponnet, 2014). Schematically, it is thought that mounting economic pressures tend to bring budgetary matters to the fore, enhancing a preoccupation with financial issues that, in many families, generates strong emotional responses in the form of depression, frustration, anger, and the like (Conger et al., 1990). In fact, apart from feeling for a partner and their sorrows and fears—which is a central part of a relationship—the most obvious channel through which an economically stressful life event of a partner is assumed to affect the mental health of the other partner is through worries about the economic situation of the couple (Bünnings et al., 2017; Marcus, 2013; Vinokur et al., 1996; Winkelmann & Winkelmann, 1995). Stress in relation to the overall debt situation in the family, for example, has been shown to explain all the effects of skipping payments (i.e., in default) on anxiety (Drentea, 2000).

Although the relation of debt issues with gender and couple processes and outcomes is still a relatively under-examined question (Dew, 2011; Eads & Tach, 2016), the family stress model—interpreted from an actor-partner perspective—assumes that financial stress crosses over from one partner to the other irrespective of gender (Ponnet, 2014), as feelings of economic pressure and perceptions of spouses overspending have been found to be associated with marital processes in the same way for men and women (Conger et al., 1999). This is, of course, not to say that the lived experience of having a financially indebted partner needs to be identical for women and men. For women, the literature, for example, has tended to focus on how they are the ones assuming the harsh work of debt management in different-sex households facing economic strain, irrespective of whether or not they are the debtor (Callegari et al., 2019; Thorne, 2010), which is in line with previous research on women’s health (Macran et al., 1996). For men, on the other hand, the focus has tended to lie on how they relate to debt in general, as something that they need to be in control of (Callegari et al., 2019; Dew & Dakin, 2011). In fact, having control over money issues has been suggested to be so ingrained in men’s image of what is expected from them that it can even be seen in how restrictive they are when it comes to accepting a partner that risks becoming a financial burden to the household in the first place (Addo, 2014).

### Aim of the Study

The objective of the present study is to determine if being prescribed antidepressants at any point in time during 2018–2019 is related to having had a different-sex spouse or cohabiting partner with a registration date for an unpaid debt in the Swedish Enforcement Authority (SEA) register during 2017. In other words, the hypothesis that I test is that financial indebtedness of a partner constitutes a risk for developing depressive morbidity for both women and men.

## Method

### Study Base: The Enforcement Authority’s Register and Other Nationwide Registers

The Swedish Enforcement Authority (SEA) is a government agency with a range of responsibilities, including, but not limited to, enforcement, debt collection, and injunctions to pay. Anybody with a legitimate payment claim can use its services, for example, the government, municipalities, companies, and private individuals (Kronofogden, 2020; Regeringskansliet, 2021). The SEA registers all debts, which have been confirmed through a simplified payment procedure or a court order and in respect of which enforcement has been attempted but has been unsuccessful (Jørgensen, 2016). An individual who has been registered at the SEA may be kept in the register for five years but can be removed earlier. In practice, a debtor is kept in the register for a minimum of three years after his or her latest matter has been closed by the Authority (SOU, 2013, p. 78).

On the basis of information extracted from the SEA database on January 11, 2018, this study focuses on the cohabiting partners or spouses of individuals, aged 20–64, who appear with a registration date for a debt in the SEA’s register dated between January 2017 and December 31, 2017. For each individual registered at the SEA, there is a set of up to five controls, drawn from the general Swedish population and matched by age, gender, and region of residence on December 31, 2014. Each of these controls has, in turn, a registered linkage, if any, to a cohabiting partner or spouse, which forms the basis for the comparison group in this study.

The data also include information from several other national registers. In this study, I use the linkages made with (1) the longitudinal integration database for health insurance and labor market studies, the total population statistics register, and the geography database and (2) the Medicinal Drug Register and the National Cause of Death Register. These registers are administered by Statistics Sweden and the National Board of Health and Welfare, respectively.

### Sample Delimitation

The study focuses on depressive morbidity in adulthood, understood as individuals between 20 and 60-years-old at baseline (2016). The individuals themselves that are followed-up for depressive morbidity did not appear in the SEA register with a registration date during the exposure year (2017) or earlier. Individuals who immigrated to Sweden in the year 2005 (date from which data from the Medicinal Drug Register is available) or later were not included in the study, nor were those who emigrated from Sweden during the follow-up period. The study is further restricted to individuals of different sex cohabiting in the same household in 2016 and registered as spouses/cohabitating partners thereinafter (2016–2019). Moreover, the study only considered persons who were alive throughout the study period and for whom complete data were available for all the variables included in the models.

A total of 4838 women and 2141 men, aged between 20–60 years, were identified as being exposed to having a different-sex spouse/cohabiting partner with a registration date for a debt in the SEA register dated between January 1, 2017 to December 31, 2017 (see Tables 1, 2, respectively). The study’s comparison group, (that is, 20–60-year-old individuals whose different-sex spouse/cohabiting partner did not appear with a registration date for a debt in the SEA register dated between January 1, 2017 to December 31, 2017), comprised 18,650 women and 11,058 men.

**Table 1:** Distribution of dependent and control variables among women, by the group that had a different-sex spouse or cohabiting partner with a registration date for a debt at the Swedish Enforcement Authority (SEA) during 2017 (exposed group) and the sample of the Swedish population (comparison group)

Variable	Female respondents		
	Exposed group		Comparison group
	Spouse/cohabiting partner registered for a debt at SEA in 2017 and still active in the register in 2018 (n = 483)	Spouse/cohabiting partner registered for a debt at SEA in 2017 but no longer active in the register in 2018 (n = 4355)	Spouse/cohabiting partner not registered for a debt at SEA in 2017 (n = 18,650)
*Dependent variable—measured in 2018 and 2019*			
Depressive morbidity (%)			
Prescribed antidepressant medication (reference category: other)	21.53	17.22	15.33
*Control variables—measured in 2016*			
Place of birth (%)			
Born in Sweden (reference category: foreign born)	73.71	86.22	88.37
Age (Mean)			
Year of birth	1976.21	1974.44	1973.37
Unemployment (%)			
Unemployed (reference category: other)	10.77	6.02	4.41
Education (%)			
Pre-upper secondary (reference category: post-upper secondary education)	13.66	5.90	4.87
Upper secondary (reference category: post-secondary education)	51.55	41.95	40.44
Form of cohabitation			
Married (reference category: unmarried)	61.49	67.14	72.51
Household type (%)			
Cohabiting without children (reference category: Cohabiting with children)	17.60	13.55	16.86
Housing tenure (%)			
Rental (reference category: ownership/tenant-owner)	39.75	13.80	11.05
Income (mean)			
Disposable household income	5205.32	7933.37	8181.78
Sickness %			
Received sickness cash benefit	20.50	17.86	17.86
*Control variable—measured during the period 2005–2016*			
History of mental illness (%)			
Prescribed psychotropic medication (reference category: other)	41.61	37.73	33.99
*Spouse/cohabiting partner’s age and region of residence – measured in 2014*			
Year of birth (Mean)	1973.55	1972.17	1971.33
Living in a big city (reference category: other) (%)	49.07	54.65	53.65

**Table 2:** Distribution of dependent and control variables among men, by the group that had a different-sex spouse or cohabiting partner with registration date for a debt at the Swedish Enforcement Authority (SEA) during 2017 (exposed group) and the sample of the Swedish population (comparison group)

Variable	Male respondents		
	Exposed group		Comparison group
	Spouse/cohabiting partner registered for a debt at SEA in 2017 and still active in the register in 2018 (n = 290)	Spouse/cohabiting partner registered for a debt at SEA in 2017 but no longer active in the register in 2018 (n = 1,851)	Spouse/cohabiting partner not registered for a debt at SEA in 2017 (n = 11,058)
*Dependent variable—measured in 2018 and 2019*			
Depressive morbidity (%)			
Prescribed antidepressant medication (reference category: other)	14.14	9.99	7.64
*Control variables—measured in 2016*			
Place of birth (%)			
Born in Sweden (reference category: foreign born)	82.76	87.95	89.64
Age (Mean)			
Year of birth	1972.47	1971.27	1971.16
Unemployment (%)			
Unemployed (reference category: other)	9.66	4.92	3.77
Education (%)			
Pre-upper secondary (reference category: post-upper secondary education)	18.97	8.91	7.76
Upper secondary (reference category: post-secondary education)	60.34	47.81	50.03
Form of cohabitation			
Married (reference category: unmarried)	59.66	70.18	72.08
Household type (%)			
Cohabiting without children (reference category: Cohabiting with children)	17.93	12.43	15.64
Housing tenure (%)			
Rental (reference category: ownership/tenant-owner)	32.07	13.24	10.94
Income (mean)			
Disposable household income	5817.90	8125.67	7303.41
Sickness %			
Received sickness cash benefit	11.03	6.86	7.61
*Control variable—measured during the period 2005–2016*			
History of mental illness (%)			
Prescribed psychotropic medication (reference category: other)	29.66	23.28	20.15
*Spouse/cohabiting partner’s age and region of residence – measured in 2014*			
Year of birth (Mean)	1975.06	1973.31	1973.42
Living in a big city (reference category: other) (%)	48.62	55.97	51.83

### Analytical Strategy

The follow-up period for the prescription of antidepressants in the exposed group is constrained to prescriptions administered within two calendar years of the partner’s registration date at the SEA, that is, the years 2018 and 2019. The comparison with the matched sample of the Swedish population is restricted to the same 2-year follow-up period. The control variables are measured at baseline for both the exposed and the comparison group (year 2016), that is, during the calendar year preceding the registration date at the SEA – except for a history of mental illness (measured during the period 2005–2016).

The information on whether a partner with a registration date, for a debt at the SEA in 2017, was still active respectively inactive for a debt at the SEA during the following calendar year is limited to one point in time (i.e., 2018-01-11). Nonetheless, the study accounts for the duration of financial indebtedness by subdividing the exposed group into two categories: (1) those with a spouse/cohabiting partner registered for a debt at the SEA in 2017 and still active in the register in 2018 and (2) those with a spouse/cohabiting partner registered for a debt at the SEA in 2017 but no longer active in the register in 2018 (see Tables 1, 2). The theoretical rationale for doing so is that even short-term changes in financial circumstances involving debt collection have been suggested to impact an individual’s mental well-being (Bond & Holkar, 2018).

**Fig. 1:**
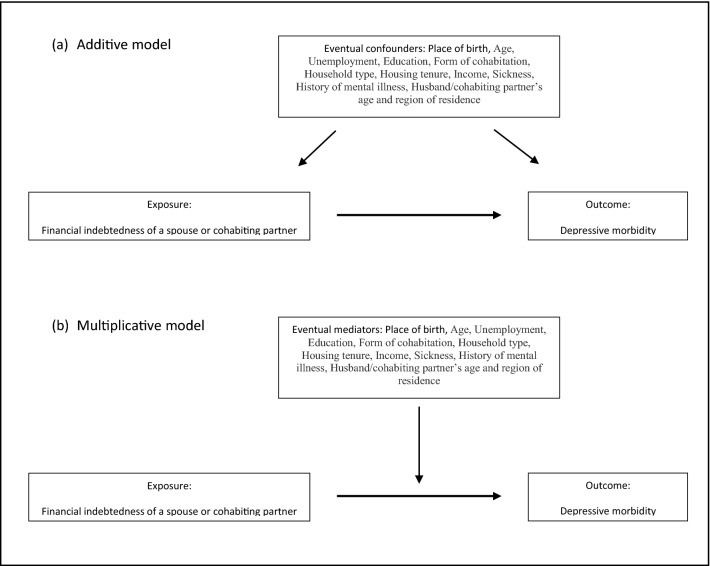
Analytical strategy

The relationship between the independent/control variables and depressive morbidity has been estimated separately for women and men, using penalized maximum likelihood logistic regression (firthlogit). The loose-matching nature of the data under study, (that is, the fact that the data have been matched on a small number of demographic variables in conjunction with the use of large sample data), allows it to be analyzed using unconditional logistic regressions (Kuo et al., 2018). The advantage of this form of analysis is that it is possible to obtain estimates for the matching variables, by simply including them as regular control variables in the analysis (in this case, the partner’s age and region of residence—both for those registered at the SEA and for those who did not appear in the SEA registry—measured on December 31, 2014). Further, firthlogit is a method that is suited for dealing with situations of possible sparse-data bias in large samples (e.g., Rojas & Stenberg, 2016), which might be the case here in the sense that the amount of prescribed antidepressants among those with a partner who was still an active debtor in the SEA in 2018 is small, and both additive (see Fig. 1a) and multiplicative (see Fig. 1b) analysis with several control variables are carried out (Cole et al., 2014). Firthlogit is accessible as a sub-routine in STATA (Firth, 1993; Hilbe, 2009; StataCorp, 2019).

The checks for interaction between the exposure variable and the statistically significant control variables were performed by including relevant product terms in the models. Although it is common practice to test for the presence of an interaction in a multiple regression (Allison, 1977), in this case, where research on the topic is still developing, it is especially important to be able to explore the extent to which the eventual detrimental effects of debts vary with the type of variables controlled for in this study (e.g., Dew, 2008).

### Dependent Variable

Depressive morbidity, understood as depressive symptoms or major depressive disorder (Silva et al., 2014), is defined as having been prescribed antidepressants (ATC-code: N06A) at any point in time during 2018–2019 (e.g., Leinonen et al., 2013). It is a dichotomous variable coded as 1 if an individual was found in the Medicinal Drug Register with a prescription of antidepressants in 2018 and/or 2019, and as 0 otherwise.

### Exposure Variable

Financial indebtedness of a partner, understood in terms of a registration at the SEA, is measured through two dummy variables. *Longer-term indebtedness:* spouse/cohabiting partner has a registration date for a debt in the SEA’s register dated from January 1, 2017 to December 31, 2017; and appear as having an active debt and/or as having a decision of debt reconstruction in the SEA’s register on January 11, 2018 (yes = 1, otherwise = 0). *Shorter-term indebtedness*: spouse/cohabiting partner has a registration date for a debt in the SEA’s register dated between January 1, 2017 to December 31, 2017; but appears as no longer having an active debt in the SEA’s register on January 11, 2018 (yes = 1, otherwise = 0).

### Control Variables

Although the knowledge concerning the extent to which an individual’s depressive morbidity can be traced to a spouse/cohabiting partner’s debt problems is limited (Callegari et al., 2019; WHO, 2011), we know a lot about how it can be traced to the individual’s own problems (e.g., Dackehag et al., 2019; Jensen et al., 2019). To be able to establish whether a partner’s registration at the SEA for a debt is associated with the other partner’s depressive morbidity, we need to control for these factors. Of special interest are those factors that are typically used to disentangle the role of debt in mental health (Bridges & Disney, 2010; Dackehag et al., 2019), such as a history of mental illness, sickness, education, income, employment and housing situations, and both individual and household demographics (Bridges & Disney, 2010; Dackehag et al., 2019).

All control variables have been measured in accordance with the analytical strategy described above and are defined as follows. *Housing tenure*: rental/ownership or tenant-owner. *Form of cohabitation*: married/unmarried. *Household type*: cohabiting without children/cohabiting with children. *Education*: pre-upper-secondary, upper-secondary, and post-upper-secondary education. *Income:* disposable household income (negative disposable income was set to zero). *Unemployment:* being registered as unemployed at the relevant authorities for at least one day over the course of a 1-year period. Sickness: having received *sickness cash benefit* from the Swedish Social Insurance Agency at least once (measured in terms of net days) over a 1-year period. *Gender*: women/men; Age: year of birth; *Region of residence*: living in one of the three regions in Sweden that includes the country’s three largest cities (Stockholm, Gothenburg, and Malmö, respectively). *Place of birth*: foreign born/born in Sweden.

*History of mental illness* is defined as having been prescribed psychotropic medications—anxiolytics (WHO Anatomical Therapeutic Chemical (ATC) code: N05B), hypnotics/sedatives (N05C), or antidepressants (N06A)—at any point in time during 2005–2016 (e.g., Jensen et al., 2019).

## Results

The study base was composed of an exposed group—that is, adults aged 20–60 with a different-sex spouse or cohabiting partner registered for a debt at the SEA in 2017—and a comparison group from the Swedish population, comprising a total of 23,488 women (see Table 1) and 13,199 men (see Table 2), respectively. The exposed group has been further subdivided into two categories: (1) those with a spouse or cohabiting partner registered for a debt at the SEA in 2017 and still active in the register on January 11, 2018 and (2) those with a spouse or cohabiting partner registered for a debt at the SEA in 2017 but no longer active in the register on January 11, 2018. This is henceforth referred to as having a spouse/cohabiting partner with longer-term indebtedness or shorter-term indebtedness, respectively.

A total of 3713 cases of prescribed antidepressants are included in the analysis of women (see Table 1). Of these prescriptions, 104 were found among those with a husband or cohabiting partner with shorter-term indebtedness; 750 among those with a husband or cohabiting partner with longer-term indebtedness; and 2,859 among those in the comparison group; that is, among those with a husband or cohabiting partner who did not appear at all in the SEA’s register on January 11, 2018, nor had a registration date for a debt at the SEA in 2017.

The proportion of depressive morbidity in the group of women with a shorter-term indebtedness is approximately two percent larger than the corresponding proportion in the group with a spouse/cohabiting partner who did not appear at all in the SEA’s register. Also, the proportion of depressive morbidity in the group of women with a spouse/cohabiting partner with longer-term indebtedness was higher than the corresponding proportion in the comparison group, about six percent (See Table 1). The distributions of the control variables clearly differ between women with a spouse/cohabiting partner with longer-term indebtedness and the comparison group, illustrating the adverse conditions of women in this life-situation (see Table 1).

In comparison, about as half as many men aged 20–60 are in the life situation of having a different-sex spouse or cohabiting partner with a registration date, for a debt, in the SEA’s register at any point during 2017 (cf., Tables 1, 2). This confirms the tendency of women having fewer problems with debts leading to a registration at the SEA. Also, the proportion of depressive morbidity in the exposed and the comparison group among men is consistently smaller than the corresponding proportions among women (cf., Tables 1, 2). However, the overall pattern of the descriptive statistics for men, presented in Table 2, is substantially the same as for women.

The results from the penalized maximum likelihood logistic regression analysis for women are presented in Table 3. In Model 1, we see that the odds of depressive morbidity differ, in a statistically significant way, by status of indebtedness of the husband/cohabiting partner. Women who had a husband/cohabiting partner with longer-term indebtedness were approximately one and a half times more likely to be prescribed antidepressants than women with a husband/cohabiting partner not registered for a debt at the SEA in 2017, nor found in the register in 2018 (OR 1.52; 95% CI 1.220–1.895).

**Table 3:** Penalized maximum likelihood logistic regression of financial indebtedness of a husband/cohabiting partner and depressive morbidity among women, aged 20–60 in Sweden 2018–2019

Variable	Model 1 Crude OR (95% CI)	Model 2 Adjusted OR (95% CI)	Model 3 Adjusted OR (95% CI)	Model 4 Adjusted OR (95% CI)	Model 5 Adjusted OR (95% CI)
*Independent variable*					
**Financial indebtedness of a husband/cohabiting partner**					
Husband/cohabiting partner Registered for a debt at the Swedish Enforcement Authority (SEA) in 2017 and still active in the register in 2018	1.52*** (1.220–1.895)	1.51*** (1.214–1.886)	1.42** (1.133–1.774)	1.31* (1.017–1.686)	1.48 (0.902–2.428)
Husband/cohabiting partner Registered for a debt at SEA in 2017 but no longer active in the register in 2018	1.15** (1.053–1.256)	1.15** (1.052–1.255)	1.14** (1.046–1.248)	1.05 (0.949–1.156)	1.24* (1.024–1.506)
(reference group: Husband/cohabiting partner not registered for a debt at SEA in 2017 nor found in the register in 2018)					
*Control variables—measured in 2016*					
Place of birth					
Born in Sweden (reference group: foreign born)			1.18** (1.054–1.327)	1.16* (1.025–1.316)	1.16* (1.026–1.318)
Age					
Year of birth			1.00 (0.991–1.002)	1.01** (1.004–1.012)	1.01** (1.002–1.014)
Unemployment					
Unemployed (reference group: other)			1.49*** (1.286–1.734)	1.21** (1.025–1.43)	1.21* (1.026–1.428)
Education					
Pre-upper secondary (reference category: post-upper secondary education)			1.23* (1.054–1.444)	0.92 (0.777–1.099)	0.92 (0.777–1.099)
Upper secondary (reference category: post-Secondary education)			1.14** (1.058–1.226)	0.99 (0.907–1.069)	0.98 (0.906–1.069)
Form of cohabitation					
Married (reference group: unmarried)			0.86*** (0.794–0.935)	0.89* (0.810–0.971)	0.89* (0.809–0.972)
Household type					
Cohabiting without children (reference group: Cohabiting with children)			0.95 (0.857–1.043)	0.91 (0.815–1.016)	0.91 (0.815–1.014)
Housing tenure					
Rental (reference group: ownership/tenant-owner)			1.18** (1.055–1.311)	1.06 (0.938–1.191)	1.06 (0.939–1.191)
Income (mean)					
Disposable household income			1.00 (1.000–1.000)	1.00 (1.000–1.000)	1.00 (1.000–1.000)
Sickness					
Received sickness cash benefit (reference group: other)				1.80*** (1.649–1.971)	1.80*** (1.649–1.971)
*Control variable—measured between 2005 and 2016*					
History of mental illness					
Prescribed psychotropic medication (reference group: other)				12.22*** (11.153–13.396)	12.82*** (11.550–14.219)
*Husband/cohabiting partner’s age and region of residence – measured in 2014*					
Living in a big city (reference group: other)		0.95 (0.886–1.019)	0.95 (0.888–1.023)	0.95 (0.876–1.027)	0.95 (0.876–1.027)
Year of birth		1.00 (0.998–1.004)	1.01 (1.000–1.011)	1.01** (1.004–1.011)	1.00 (0.998–1.009)
*Interaction terms*					
History of mental illness * Spouse/cohabiting partner still active at SEA History of mental illness * Spouse/cohabiting partner no longer active at SEA					0.86 (0.484–1.514) 0.80* (0.638–0.998)
Prescribed antidepressants	3,713	3,713	3,713	3,713	3,713
Total study population (n)	n = 23,488	n = 23,488	n = 23,488	n = 23,488	n = 23,488

*p < 0.05, **p < 0.01, ***p < 0.001

The corresponding odds of depressive morbidity for women with a husband/cohabiting partner with shorter-term indebtedness compared to women with a husband/cohabiting partner not registered at the SEA was 1.15, which was also statistically significant (95% CI: 1.053–1.256).

As can be seen in Model 2 (Table 3), this relationship does not change when adjusted for husband/cohabiting partner’s age and region of residence, nor when women’s own age, place of birth, employment status, educational level, form of cohabitation, housing tenure, or income are adjusted for in Model 3. The odds of depressive morbidity still differ, in a statistically significant way, by status of indebtedness of the husband/cohabiting partner (OR 1.42 and OR 1.14, respectively).

In Models 4 and 5 (Table 3), sickness and history of mental illness are introduced in the analysis, resulting in an interaction effect between financial indebtedness of a husband/cohabiting partner and history of mental illness in relation to depressive morbidity. However, only the interaction between having been prescribed psychotropic medication between 2005 and 2016 and having a husband/cohabiting partner with shorter-term indebtedness is statistically significant (see interaction terms in Model 5, Table 3). This indicates that within the shorter-term indebtedness group, only women without a history of mental illness are at higher risk of depressive morbidity in this group (cf., OR 1.05 and 95% CI 0.949–1.156 in Model 4 with OR 1.24 and 95% CI 1.024–1.506 in Model 5, Table 3). The odds of depressive morbidity for women who had a husband/cohabiting partner with longer-term indebtedness are still statistically significantly higher (OR 1.31; 95% CI 1.017–1.686) compared to women with a husband/cohabiting partner not registered for a debt at the SEA in 2017 nor found in the register in 2018, irrespective of a history of mental illness (see the not statistically significant interaction term in Model 5, Table 3). No other tested interaction between the independent variable and statistically significant control variables in the final model showed to be significant.

The results from the penalized maximum likelihood logistic regression analysis for men are presented in Table 4. In general, the pattern for men mirrors that of women’s (cf., Table 4 and Table 3) up until Model 4. The odds of depressive morbidity among men also differ in a statistically significant way, by status of indebtedness of the spouse/cohabiting partner, but it does not interact with any of the significant control variables introduced in the final model (Model 4, Table 4). However, beyond the difference that the higher odds of depressive morbidity among men with a wife/cohabitating partner with shorter-term indebtedness does not vary with history of mental illness, the final Odds Ratios found for men between the status of indebtedness and depressive morbidity (OR 1.57 [95% CI 1.072–2.307] and OR 1.29 [95% CI 1.071–1.552] in Model 4, Table 4) seem to be of the same order of magnitude as the statistically significant ones found among women (OR 1.31 [95% CI 1.017–1.686] in Model 4, Table 3; and OR 1.24 [95% CI 1.024–1.506] in Model 5, Table 3; respectively).

**Table 4:** Penalized maximum likelihood logistic regression of financial indebtedness of a wife/cohabiting partner and depressive morbidity among men, aged 20–60 in Sweden 2018–2019

Variable	Model 1 Crude OR (95% CI)	Model 2 Adjusted OR (95% CI)	Model 3 Adjusted OR (95% CI)	Model 4 Adjusted OR (95% CI)
*Independent variable*				
**Financial indebtedness of a wife/cohabiting partner**				
Wife/cohabiting partner Registered for a debt at the Swedish Enforcement Authority (SEA) in 2017 and still active in the register in 2018	2.01*** (1.436–2.812)	2.04*** (1.456–2.852)	1.80** (1.280–2.532)	1.57* (1.072–2.307)
Wife/cohabiting partner Registered for a debt at SEA in 2017 but no longer active in the register in 2018	1.35** (1.138–1.589)	1.34** (1.134–1.584)	1.34** (1.134–1.587)	1.29** (1.071–1.552)
(reference group: Wife/cohabiting partner not registered for a debt at SEA in 2017 nor found in the register in 2018)				
*Control variables—measured in 2016*				
Place of birth				
Born in Sweden (reference group: foreign born)			1.21 (0.978–1.499)	1.38** (1.099–1.741)
Age				
Year of birth			0.98*** (0.975–0.990)	1.01 (0.991–1019)
Unemployment				
Unemployed (reference group: other)			1.29 (0.969–1.714)	1.02 **(**0.746–1.394)
Education				
Pre-upper secondary (reference category: post-upper secondary education)			1.28* (1.019–1.611)	1.04 (0.811–1.341)
Upper secondary (reference category: post-secondary education)			1.18* (1.027–1.349)	1.09 (0.939–1.264)
Form of cohabitation				
Married (reference group: unmarried)			0.92 (0.798–1.068)	0.96 (0.812–1.125)
Household type				
Cohabiting without children (reference group: Cohabiting with children)			0.92 (0.781–1.088)	0.96 (0.796–1.156)
Housing tenure				
Rental (reference group: ownership/tenant-owner)			1.46*** (1.215–1.753)	1.28* (1.046–1.566)
Income (mean)				
Disposable household income			1.00 (1.000–1.000)	1.00 (1.000–1.000)
Sickness				
Received sickness cash benefit (reference group: other)				2.10*** (1.739–2.525)
*Control variable—measured between 2005 and 2016*				
History of mental illness				
Prescribed psychotropic medication (reference group: other)				14.73*** (12.678–17.106)
*Husband/cohabiting partner’s age and region of residence – measured in 2014*				
Living in a big city (reference group: other)		1.06 (0.935–1.201)	1.08 (0.952–1.230)	1.00 (0.873–1.155)
Year of birth		0.99* (0.990–0.995)	1.01* (1.001–1.017)	1.00 (0.979–1.014)
Prescribed antidepressants	1,071	1,071	1,071	1,071
Total study population (n)	n = 13,199	n = 13,199	n = 13,199	n = 13,199

* p < 0.05, ** p < 0.01, *** p < 0.001

## Discussion

To the best of my knowledge, this study is the first to examine depressive morbidity in relation to financial indebtedness of a cohabiting partner or spouse, using large-scale register data for an entire country (Dackehag et al., 2019; Richardson et al., 2013; Turunen & Hiilamo, 2014). This has made it possible, in an unprecedented way, to measure the relation using the so-called objective indicators (i.e., register information for prescription of antidepressants and unpaid debt, respectively) and control for crucial factors that may have been confounding the relationship, including a history of mental illness and other economic stressors (such as unemployment). The main result of the study, (that is, depressive morbidity being significantly related to the financial indebtedness of a cohabiting partner adjusted for multiple background variables), is in line with the theoretical assumptions outlined at the outset of the study, as well as with recent calls from international authorities to be aware of the possibility that the impact of unfavorable financial changes may very well go beyond the directly affected individual within a family (WHO, 2009). However, there are important specificities in the results that need to be addressed.

The fact that debt counseling seems to have a positive effect on mitigating the unhealthy, stressful impact of being unable to meet one’s financial commitments (Turunen & Hiilamo, 2014; WHO, 2011)—in conjunction with research suggesting women as being the ones engaging in the harsh work of debt management even when the debt is not theirs (Callegari et al., 2019)—has recently prompted scholars to call for the need for social services to be attentive to the life situation of an eventual female partner when providing service to men with debt problems (Callegari et al., 2019). In Sweden, it is the municipal social services that are expected to provide budget and debt advice to indebted people—both to prevent over-indebtedness and to help them find a solution to their problems, in accordance with the Social Services Act (SoL) (SFS, 2001:453, 5 chapter 12 §). The results of this study give support to current concern for women’s situation, as they show that there were considerably more women than men who found themselves exposed to having a spouse or cohabitating partner being registered at an enforcement authority for an unpaid debt—in Sweden, in 2017—and that living under this condition may have negative consequences for the individual’s mental health, at least in terms of depressive morbidity.

The results also suggest that it is important not to lose sight of the fact that although men as husbands/cohabiting partners as a group may, in terms of quantity be less afflicted by the actual problem, they do suffer the consequences of financial indebtedness of a wife/cohabiting partner. In other words, there is no indication that the negative effect of living under this condition would be very much different for men. In fact, if anything, men’s mental health would seem to be somewhat more compromised, considering that having a history of mental illness did not prevent them from being at a higher risk of developing depressive morbidity given a wife/cohabiting partner’s difficulties in meeting her financial commitments. Hence, the current gender-aware approach that is being advocated for programs dealing with indebted clients within the social services in order to come to terms with women’s exposed situation in indebted household—including discussing the households’ decision-making processes, division of financial responsibility, financial standards for all individuals in the household, and encouraging both parties of a household to take part in the intervention process (Callegari et al., 2019)—needs to be sensitive to these results and also carefully articulate how this approach should be implemented when men are not the ones with the problematic debt in the couple relationship. In particular, considering that the literature, although still rather speculative (Dew & Dakin, 2011), is suggesting that men’s expectation of being in control regarding money makes them relate to financial disagreements in a relationship in a way that women do not.

Theories on the relation between debt issues and couple processes (gender included) and outcomes are still relatively underdeveloped (Dew, 2011; Eads & Tach, 2016). Although the main point of departure for this study has been the family stress model’s basic assumption of financial strain—where experiencing difficulties paying bills is just one of the many indicators of this—being related to the emotional distress of both parties in a couple, the results of this study fall well within recent attempts to articulate debt changes as a phenomenon that in itself is worthy of analysis in the dynamics of family relations (e.g., Dew, 2008). Of special interest in this respect is that financial indebtedness was significantly related to depressive morbidity above and beyond other conditions of economic strain (e.g., unemployment). Furthermore, the condition where a household falls behind in its loan payments and being unable to escape the legal consequences of unmet financial obligations, ultimately leading to depressive morbidity, can very well be seen as part of the other end of the continuum of debt changes within a family; that is, the satisfaction that comes from becoming debt free (e.g., Dew, 2008).

The results of this study also indicate that both shorter and longer spells of financial indebtedness of a partner are related to the mental health of the other partner, with the reservation that for women the detrimental impact of shorter-term indebtedness of a partner was only significant among those with no prior history of mental illness. This is in line with previous studies suggesting that even shorter-term changes in financial circumstances involving debt collection may have a detrimental impact on an individual’s mental health (Bond & Holkar, 2018). After all, it takes time to recuperate from financial hardship, for example, the note of payment default added to the debtors’ credit record in connection with a decision on debt repayment can be kept for year. In short, the worries about the economic situation of the couple that has been assumed to connect the economically stressful life event of indebtedness of the partner to the mental health of the other partner (Bünnings et al., 2017; Marcus, 2013; Vinokur et al., 1996; Winkelmann & Winkelmann, 1995) are likely to continue, at least to some degree, even after a partner that is no longer active for a debt at an enforcement authority.

With large sample data, it is quite possible to have a statistically significant finding from a weak but true association between a risk factor and a disease (Chen et al., 2010). Although the size of the odds ratios found in this study for the relationship between an individual’s depressive morbidity and the status of financial indebtedness of their spouse/cohabiting partner may be interpreted as small, they are far from negligible (cf., Ialongo, 2016). There are at least four main reasons for interpreting the odds ratios in question in this way: 1) they remained statistically significant, controlling for a range of relevant circumstances pertaining directly to the individual; (2) they were in the same order of magnitude, in terms of size, as the statistically significant odds ratios of other risk factors (e.g., unemployment for women and housing tenure for men); (3) they are theoretically founded—for example, by the family stress model (Conger et al., 1990; Ponnet, 2014); and (4) they follow empirical patterns from adjacent research traditions, showing a cross-over effect within couples in the presence of diverse socio-economic stressors, for example, cross-over effects of employment insecurity (Inanc, 2018).

By contextualizing the found odds ratio in this way (Ialongo, 2016), one could argue that these results constitute an important contribution to the evidence base of a relation that although expected has hitherto tended to be hidden, not the least, in the analysis of the aftermath of the latest global financial debt crisis (Turunen & Hiilamo, 2014; WHO, 2011). Future assessments of broad and multifaceted antipoverty programs, including everything from asset transfers, cash support, skills training, and access to savings opportunities—that seem to have a substantial positive impact on an individual’s mental health (e.g., increase in happiness and reduction in depression, stress, and worries) (Ridley et al., 2020)—ought to consider that there may be even more positive impacts, in the form of the so-called spillover effects to others than the directly targeted individual (cf., Baranowska-Rataj & Högberg, 2018). If such a link could be established, the legitimacy of social and economic policies, in the efforts to come to terms with this complex phenomena, would be further strengthened (Jacob, 2012).

## Limitations

Three main methodological considerations should be considered when interpreting the results of this study. Firstly, because the study is entirely based on register data, there is a lack of self-reported information on confounders that may have influenced the results (Thygesen & Ersbøll, 2014), for example, relationship satisfaction (Roy, 1987; Vinokur et al., 1996). However, capturing representative and sizeable groups of individuals with a spouse/cohabiting partner with debt problems through traditional surveys and following them up in terms of mental health has thus far proven to be very difficult (Richardson et al., 2013; Turunen & Hiilamo, 2014; Webley & Nyhus, 2001). Thus, register-based studies of the kind presented here are very important (Thygesen & Ersbøll, 2014).

Secondly, this study is limited to depressive morbidity measured as a registration of prescription of antidepressants in the Medicinal Drug Register, that is, to a status of mental illness that not only presupposes that the individual has met a physician (i.e., health seeking behavior), but also that the physician detected the mental illness in question and prescribed a drug treatment for it. In practice, this restricts the degree to which the results can be extrapolated beyond the confines of these specific types of registered illness. Far from all cases of depressive morbidity in a population come to the attention of the authorities; for example, it has been estimated that the treatment gap (i.e., a measure of how many individuals with psychiatric disorders remain untreated although effective treatments exist) for major depression in the WHO European Region is 45.5% (Kohn et al., 2004).

Finally, as with all single-country studies (Pepinsky, 2019), the cross-over effects of financial indebtedness on depressive morbidity found here may not be generalizable to other countries. It has for example been suggested that cultural differences could play a role in how people respond to debt repayment problems (Lester & Yang, 2012). Having said that, the Swedish legal system for countering financial indebtedness is not unique, similar ones can, for example, be found in the other Nordic countries, Great Britain and The Netherlands (SOU, 2013, p. 78), suggesting that the results from this study may be of value beyond the boundaries of Sweden.

## Conclusion

An individual’s mental health is directly related to the status of financial indebtedness of a different-sex spouse/cohabiting partner. Both women and men are at an increased risk of suffering from depressive morbidity if the spouse/cohabiting partner remains active for a debt at the SEA within a one-year period, irrespective of their own health, employment, socioeconomic status, and other background variables. The results give support to current calls to be attentive to the plausible, albeit rather hidden, detrimental health impact that financial indebtedness has on others, than the directly affected individual.
